# Supplementary material for: Long-Term Influence of Locus of Control and Quality of Life on Metabolic Profile in Elderly Subjects with Type 2 Diabetes
Source: Int J Environ Res Public Health. 2022 Oct 17;19(20):13381. doi: 10.3390/ijerph192013381 (PMC9602557; doi:10.3390/ijerph192013381)
Supplement: Supplementary file 1 [file ijerph-19-13381-s001.zip › ijerph-1921730-supplementary.pdf]

Supplemental Table S1. Change from baseline in study variables during the 6 years-follow-up, according to gender.

**Variable**	**Change from baseline**	**Women n=41**	**Men n=63**	**p**
Weight (Kg)	-0.02 ± 0.066	-0.01 ± 0.06	0.14 ± 1.24	0.36
BMI (Kg/m^2)	-0.02 ± 0.063	-0.01 ± 0.05	-0.03 ± 0.06	0.13
HbA1c (%)	-0.004 ± 0.10	0.01 ± 0.10	-0.01 ± 0.11	0.27
Patients with HbA1c ≤7.0% (%)	-17.3%	-14.6%	-19.0%	0.54
eGFR (ml/min/1.73 m^2)	-0.015 ± 0.29	-0.02 ± 0.25	-0.005 ± 0.32	0.71
Subjects with chronic complications n (%)	+38%	+26.8%	+ 28.6%	0.89
Intensification of diabetes therapy (%)*	+30	+31.6%	+26.9%	0.79

Data are mean ± SD; *Intensification of diabetes therapy: increase in the number of OHA or addition of injection drugs (insulin or GLP-1 RAs).

Supplemental Table S2. Tests scores at baseline, in T2D subjects developing or not developing new chronic complications.

**Clinical baseline characteristics**	**Subjects developing new chronic complications**	**Subjects not developing new chronic complications**	**p**
Mini Mental State Examination (MMSE)	24.71 ± 2.79	26.63 ± 2.91	0.07
ADL (Activities of Daily Living)	5.79 ± 0.71	5.81 ± 0.49	0.058
IADL (Instrumental Activities of Daily Living)	7.63 ± 0.89	7.67 ± 0.93	0.065
Diabetes Treatment Satisfaction Questionnaire			
total score	31.81 ± 5.11	32.34 ± 4.94	0.08
DTSQ 2	2.7 ± 1.77	3.0 ± 1.83	0.20
DTSQ 3	1.6 ± 1.66	1.6 ± 1.79	0.058
Diabetes Specific Quality of Life (DSQoL)			
Satisfaction	33.16 ± 9.38	30.3 ± 9.08	0.17
Impact	37.51 ± 8.23	35.15 ± 9.02	0.21
Worry	8.36 ± 2.88	8.19 ± 2.97	0.78
Locus of Control (LOC)			
Internal	29.05 ± 6.48	30.89 ± 5.78	0.19
External	24.52 ± 5.69	24.96 ± 5.93	0.73
Chance	18.21 ± 7.62	18.37 ± 8.09	0.92

Data are mean ± SD;

DTSQ 2: perceived hyperglycaemia; DTSQ 3: perceived hypoglycaemia;

MMSE: Mini Mental State Examination; DSQoL: Diabetes Specific Quality of Life LOC: Locus of Control; DTSQ: Diabetes Treatment Satisfaction Questionnaire.

Supplemental Figure S1. Box plots for the association of good metabolic control maintenance with baseline HbA1c and LOC External.

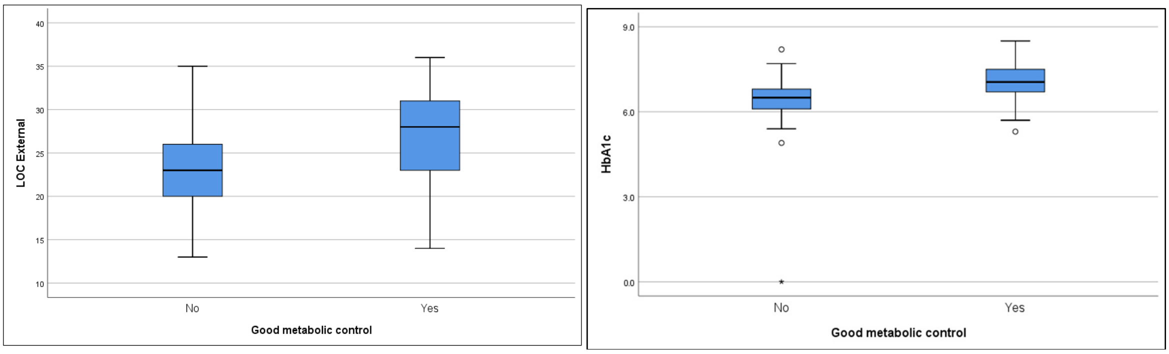

LOC: Locus of Control.
